# Dental Academic Degrees in Germany Compared to the USA

**DOI:** 10.3390/dj10060098

**Published:** 2022-06-02

**Authors:** Nikoletta Vargas, Georgios E. Romanos

**Affiliations:** 1 Department of Dentistry, Albert Einstein College of Medicine, New York, NY 10461, USA; nikoletta.vargas@alum.urmc.rochester.edu; 2 Department of Periodontology, School of Dental Medicine, Stony Brook University, Stony Brook, NY 11794, USA; 3 Department of Oral Surgery and Implant Dentistry, Dental School (Carolinum), Johann Wolfgang Goethe University, 60596 Frankfurt, Germany

**Keywords:** dental education, dental academic degrees, doctoral degrees, postgraduate education

## Abstract

There are different avenues for obtaining postgraduate doctoral/Ph.D. degrees in Germany and abroad. Depending on their interests and career plans, candidates can choose a postgraduate doctorate/Ph.D. that focuses on a career in academia or a doctorate that does not involve all elements of a Ph.D. and is obtained for the title’s sake. Germany offers this type of diversity and flexibility, whereas the USA postgraduate doctorate model presents a more structured doctorate. The current article provides insight regarding various and more flexible pathways for obtaining a postgraduate doctorate by comparing the German and the American model. The diversity of academic degrees in dentistry and medicine, such as postgraduate doctoral degrees and the higher postdoctoral degrees available in Germany for graduates interested in academia, makes educational evaluation processes and credentials recognition challenging. The lack of transparency and a systematic approach for the academic acknowledgment of the different scientific values of each doctorate type is creating confusion, primarily when German postgraduate doctorate holders pursue academic careers internationally. The current article aims to enhance the knowledge about the different academic degrees and facilitate the educational evaluations, specialty applications, and employment processes. Understanding the additional scientific value of each doctorate type offered in Germany is imperative for their credential recognition internationally.

## 1. Introduction

Education plays a crucial role in our modern life. Higher education is becoming more attractive for young professionals, as it provides a higher standing in the hierarchy and opens doors for academic and financial opportunities. It is becoming more critical for students already in their careers to think about higher education. However, the number of graduates worldwide who pursue postgraduate education is increasing exponentially due to the high demand for different scientific disciplines. Modern technologies and newly emerging approaches require highly qualified professionals. Ambitious young graduates have various options after graduating from dental schools in Germany. However, individuals interested in an academic career are required to obtain higher postgraduate education after they obtain their university degree and qualifications and requirements for incorporating teaching experience. Dental and medical students in some European countries, such as Poland, Slovenia, Bulgaria, England, Austria, as well as Russia, and many English speaking countries receive the university degree DMD/DDS/MD after graduation, which gives the title of “doctor” as a courtesy title, leading to a lower proportion of student research in those countries because the dental/medical students do not necessarily have to obtain a postgraduate doctoral degree to be called “doctor” [1]. In Germany, students receive a university degree Zahnarzt/Arzt for dentistry and medicine, respectively. Although these degrees have the same value as the DMD/DDS/MD degree of English-speaking countries, they do not contain the title of “doctor.” Therefore, German dental and medical students are forced to pursue postgraduate doctoral degrees to be officially called “doctor”. Germany’s rules are strict regarding these guidelines, as dentists and physicians are not allowed to call themselves “doctor” if they have not obtained a higher postgraduate degree.

**Figure 1:**
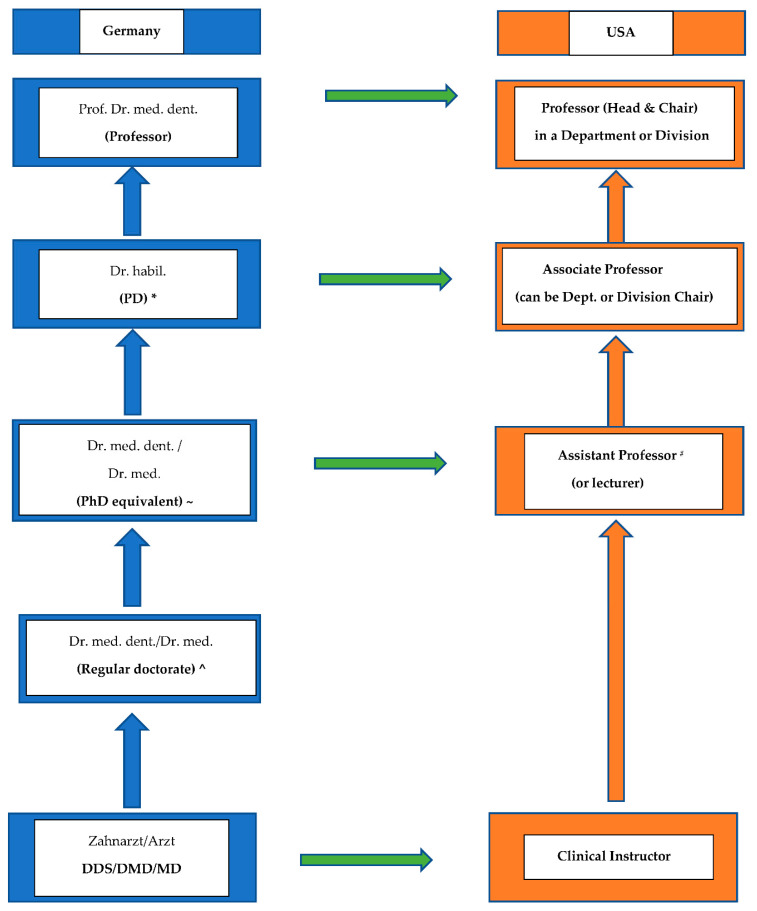
German Dental and Medical Academic Rankings in relationship to the U.S. system. ^ mostly focused on clinical career, can pursue academic or clinical career (lower scientific value, lower grades (rite/cum laude)); ~ preferred doctorate type due to the possibility for higher grades (magna/summa cum laude); * PD—Privatdozent, only in case of affiliation and teaching within the university; ^#^ might be the senior lecturer/Assistant Professor (“Oberarzt”) and must have the doctoral (Dr. med. dent.) but not necessarily the postdoctoral degree (so-called “Habilitation”).

There is confusion about the level of postgraduate academic education in various countries. A more systematic approach to requirements and standards in regard to dental academic degrees is essential for a better and more accurate evaluation of equivalent academic degrees internationally due to today’s scientific globalization. We need to differentiate the experiences and degrees of postgraduate clinical advanced standing programs and specialty training from solid scientific academic training to completing an academic postgraduate degree. Some of these degrees become a minimum requirement to be hired in some European countries for specific academic positions. The knowledge about the different academic degrees can simplify the process of employment status, recruiting competent, proficient physician-scientists with adequate experience and qualifications, which can further improve the level of education internationally. Dentists in the USA graduate with the DMD or DDS degree (dependent on the school) and the title of Doctor of Medicine in Dentistry or Doctor of Dental Surgery, which has the same educational and clinical requirements equivalent to the European standards for clinical and educational requirements, and the obtained university degree is considered equivalent. To obtain the DMD or DDS status, a postgraduate doctoral degree is not required. However, there are differences in the two systems (the European and the U.S.) in terms of qualifications in the hiring processes and requirements for doctoral degree completion to enter higher education at the rank of an Assistant Professor (Figure 1).

There is a conflicting distinction between the academic dental degrees and titles in Germany. It is of significant value for young dentists who want to establish their careers and grow academically to recognize and understand the pathways and exact avenues for establishing their goals. The first degree available in the academic hierarchy after graduating from dental school across the globe is a master’s (MS), followed by a doctoral or Ph.D. degree. Academic growth and promotion are associated with the continuous contribution to science. Indeed, there are continuing education courses in the postgraduate level for professional development, without certificates recognizing the educational experience or competency of the candidates.

In contrast to the academic system of English-speaking countries, such as the USA and Canada, where Ph.D. programs are more structured (associated with extensive duration and a relative lack of flexibility), the German academic system offers several different options for obtaining a postgraduate doctoral degree depending on the candidate’s needs and future plans, regardless of in which scientific field and profession. Dental students often prefer research topics for their Ph.D. unrelated to dentistry (such as neurophysiology, immunology, infectious diseases, etc.) and can choose among two types of postgraduate doctorates. Less is known about the actual differences of these dental academic degrees in terms of scientific value. Most published studies evaluating the German postgraduate doctoral degrees in healthcare focus on medicine. They do not provide sufficient information about the pathways for obtaining a higher doctoral degree in dentistry, most likely because both fields overlap and reveal identical problems and characteristics and similar criticisms [2,3,4,5]. The present article highlights different avenues to postgraduate doctoral and postdoctoral degrees in Germany. It compares the academic systems with English-speaking countries, in particular the USA, which makes the evaluation and credential processes more transparent. The options are similar in medicine and dentistry, but the authors focus on the dental degrees and academic rankings for promotion.

## 2. Postgraduate Doctoral Degrees/Ph.D. in the USA

Depending on funding, universities in the United States of America can be public or private. The level of education and requirements are similar in both groups; however, the academic ranking and tuition fees remain factors for students’ choice. The main difference that directly affects postgraduate doctorate/Ph.D. students is tuition. Private universities are more expensive; however, they maintain a similar tuition fee for local state students, U.S. citizens, green card holders, and international students. In contrast, tuition in public universities almost always varies among these categories of students. In general, postgraduate doctorate/Ph.D. programs in the USA are structured and involve a rigorous didactic element, such as core and elective classes during the first half of the program. Once these courses are completed, Ph.D. students have to undergo a comprehensive examination, also known as “field exam” or “dissertation qualifying exam”, to demonstrate profound knowledge in the field of their interest and ability to conduct their own research. The usual timeline for doctorate/Ph.D. programs is 4 to 6 years, with the scientific research in the second part of the program. Ph.D. students can choose from traditionally structured Ph.D. programs or integrated Ph.D. programs, the latter being especially preferred by medical and dental students. Most of the published data about Ph.D. programs in healthcare professions in the USA are focused on nursing or medicine. The need for evidence regarding the trends in Ph.D. programs in dentistry is increasing exponentially. The available data on integrated MD–Ph.D. programs focus on the benefits and time management for young students interested in a career in academia and who want to become “physician-scientists”, also known as “physician-investigators.” The integrated MD–Ph.D. programs were established to realize that the standard four-year medical school curriculum is neither intended nor sufficient to train physician-scientists to be proficient in clinical medicine and scientific research [6]. MD–Ph.D. programs have become very popular in recent years, and dental schools are following a similar trend; however, the number of applicants to these programs each year remains a small fraction of the total number of medical/dental school graduates. The need for flexibility and efficiency today in our modern times is making these integrated MD–PhD/DDS–Ph.D. programs a valuable option for young academia-oriented future “physician-scientists”. They offer the advantage of starting with the didactic and part of the scientific research before graduating from medical/dental school, which is very similar to the German academic model. More clarity and input to motivate young students and peak their interest for scientific research is necessary. There are many internet resources with information for prospective students. Many universities offer exclusively online Ph.D. programs. However, the official sources, such as the U.S. Department of Education https://www.ed.gov (accessed on 14 March 2022), National Institutes of Health https://www.nih.gov (accessed on 14 March 2022), the National Postsecondary Education Cooperative (NPEC) http://nces.ed.gov/npec (accessed on 14 March 2022), and the National Center for Education Statistics (NCES) http://nces.ed.gov (accessed on 14 March 2022), remain the most trusted sources of information, which provide the most accurate data and statistics regarding postgraduate education on the internet. 

## 3. Postgraduate Doctoral Degrees/Ph.D. in Germany

Almost all universities in Germany are public institutions. Due to their federal structure, German universities are financed and controlled by the respective Federal Ministries of Education. The Acts of Higher Education of each federal state regulate the universities, and their relationships with the relevant Ministry of the federal state and are coordinated by the Framework Act of Higher Education (Hochschulrahmengesetz) (https://www.eui.eu/ProgrammesAndFellowships/AcademicCareersObservatory/AcademicCareersbyCountry/Germany (accessed on 10 December 2021)).

This article presents a thorough review of the information provided by the most trusted sources, among which are the German Federal Ministry of Education and Research and the DAAD (Deutscher Akademischer Austauschdienst, German Academic Exchange Service). According to these sources, almost 29,000 doctoral candidates earn a doctorate degree/Ph.D., also known as “Promotion” (German), per year in Germany. Cort-Denis Hachmeister describes the distribution of the doctorates in the different scientific fields in his article from 2019 “Promotionen als Indikator für die Leistung von Hochschulen Auswertung von Daten des Statistischen im Blickpunkt” (https://www.che.de/download/im_blickpunkt_promotionen_2019-pdf/ (accessed on 10 December 2021)). According to data from the CHE Ranking (Centre for Higher Education) and from the German Federal Statistical Office, the awarding of doctorate degrees is most prominent in medicine and the natural sciences. In the time frame between 2015 and 2017, a total of 6274 doctoral degrees were awarded in medicine, 2498 in biology, 915 in dentistry, 485 in veterinary medicine, and 391 in pharmacology. According to the collected data, around half of the graduates in medicine and dentistry proceed with a postgraduate doctoral degree. Numerous other doctoral degree holders belong to fields that do not have anything in common with medical sciences, such as architecture (100), sport sciences (114), mathematics (615), and computer sciences (946). A total of 29,303 doctoral degrees for the year 2016 according to the Organization for Economic Co-operation and Development OECD 2016, (Table 1) were awarded in Germany. 28,404 in 2017, 27,838 in 2018, and 28,690 in 2019, respectively, according to the official website of the Statistisches Bundesamt, DESTATIS (https://www.destatis.de/DE/Themen/Gesellschaft-Umwelt/Bildung-Forschung-Kultur/Hochschulen/Tabellen/promotionen-bundeslaender.html (accessed on 10 December 2021)). According to their data, the prevalence of female doctoral degree recipients is slightly increasing—13,038 out of 28,690 (in 2019) compared to 12,577 out of 27,838 (in 2018), and 5199 vs. 5688 of these were individuals from abroad who earned their degrees at German universities, respectively. The official website of the German Federal Ministry of Education and Research provides detailed information about the different postgraduate doctoral degree/Ph.D. pathways. It describes all available options for German and international postgraduate candidates for academic career paths. This is one of the most trusted websites, as it is an online information resource provided directly by the German Federal Ministry of Education and Research. It helps young academics and professionals who wish to pursue an academic career to be well informed about the various options (s. also: https://www.research-in-germany.org/en/jobs-and-careers/info-for-phd-students.html (accessed on 10 December 2021)). According to the information provided by this official online source, Germany is considered one of the world’s most attractive research and higher education nations. Nearly 412,000 international students pursue higher education in Germany, more than 5600 international students complete their doctoral degrees annually, and approximately 50,000 international professionals work in German higher education institutions.

Almost 115,000 out of 434,000 research and development projects are completed at higher education institutions and University hospitals in Germany. Furthermore, such institutions offer a broad spectrum of research activities for students and doctorate holders, including basic research and applied research and development. The types of postgraduate doctoral degree/Ph.D., based on the German Federal Ministry of Education and Research official website, can be related to the doctoral degrees medical and dental students pursue after graduation (https://www.research-in-germany.org/en/your-goal/phd/two-ways-to-get-your-phd.html (accessed on 15 March 2022)).

Following the trends of social media due to its efficiency in spreading information and effectiveness in awakening the interest for scientific research among more young people, the German Federal Ministry of Education and Research also embedded a YouTube video on their official internet website and on their YouTube Channel. This video helps further clarify the differences, similarities, and mutual goals of the different pathways for postgraduate education and Ph.D. completion in Germany. It is a very well-done animated summary, which helps individuals interested in obtaining a postgraduate Ph.D. degree visually understand the similarities, differences, and the emphasis on the one and same goal—a postgraduate academic doctorate/Ph.D. degree (https://www.youtube.com/watch?v=os-Ymod7_oc&t=110s (accessed on 15 March 2022)).

The pathways for obtaining a postgraduate doctoral degree/Ph.D. are thoroughly described on the official internet website of the German Federal Ministry of Education and Research. They can be related to the types of postgraduate doctoral degrees dental and medical students pursue in Germany.

## 4. Types of Postgraduate Doctoral Degrees/Ph.D. in Germany

According to the German Federal Ministry of Education and Research, there are three pathways for obtaining a postgraduate doctoral/Ph.D. degree in Germany:

1. Traditional, Individual doctoral degree/Ph.D.;
2. Structured doctoral degree/Ph.D. program;
3. Doctorate/Ph.D. in cooperation with a company.

**Figure 2:**
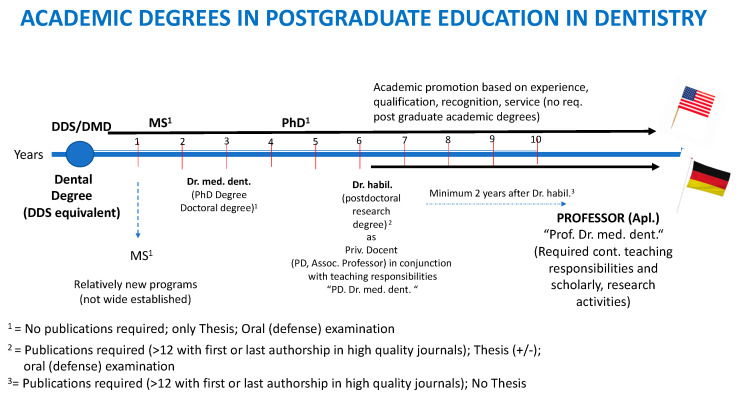
Academic Degrees and Postgraduate Education in Dentistry.

Depending on an individual’s interests, the two most desirable tracks to obtain a doctoral degree/Ph.D. at a German University with flexibility and availability are the traditional, individual doctorates/Ph.D., and/or the structured doctorate/Ph.D. programs. The traditional, individual doctorate/Ph.D. has been the “gold standard” and the most common type of study in the field of medicine and dentistry in Germany. In recent years, several German universities are beginning to consider offering structured Ph.D. programs following the academic model of English-speaking countries (UK or USA). These structured Ph.D. programs require around three more additional years of study after graduation (i.e., after the 6 years of studies in medicine or five years in dentistry) (Figure 2). In contrast to the structured Ph.D. programs, the traditional, individual doctoral degree/Ph.D. can be initiated during the undergraduate studies of dentistry (or medicine) but must be completed after graduation, which is the reason why it is considered a postgraduate doctoral degree. These traditional doctoral degrees are dependent on a variety of factors, such as the complexity of the scientific project, efforts, time invested, and successful completion, and they usually take at least one or more years after graduation to obtain the doctoral degree. The third option for a postgraduate doctoral degree/Ph.D. in Germany involves a collaboration with a company, but this option is not very popular among medical and dental postgraduate students.

## 5. Traditional, Individual Doctorate 

According to the German Council of Science and Humanities (Wissenschaftsrat), 93% of the total postgraduate doctoral degree/Ph.D. holders complete a traditional doctorate; the other 7% choose structured programs. One of the reasons why the traditional, individual doctorate degree is preferred, especially in the basic natural sciences and health professions, is the opportunity for employment in the candidates’ fields simultaneously. It is based on rigorous, extensive scientific research carried out independently or under the supervision of one professor (supervisor, mentor), also known as “Doktorvater”/“Doktormutter”. This pathway to a doctoral degree remains the most common avenue in Germany and Switzerland. Additionally, for the extensive independent scientific research approach with data collection and analysis, there is a requirement for the completion of a thesis (dissertation) under the supervision of a mentor. The dissertation can be written in German or English, based on mutual agreement between the candidate and the supervising professor at the beginning of the scientific project and officially determined in the doctoral contract. Once the dissertation is submitted and accepted by a commission, the candidate is expected to appear for an oral defense examination in front of a committee. This is the traditional path, which is pursued by the majority (over three-quarters of all doctoral students in Germany). This postgraduate doctoral degree/Ph.D. type is very common because it gives candidates great flexibility. They can be employed simultaneously in their field and work on their doctorate after work hours, on weekends, and on holidays. Usually, the research and the thesis must be completed within 5 years from the date the applicant applied to start the doctoral degree process and is dependent on the promotion regulations determined by the university. The same principles apply for medicine and dentistry. Dental students can choose between two types of individual doctorates: (a) regular doctorate, Dr. med. dent., and (b) research (focused)-intense Dr. med. dent./Ph.D. equivalent. The current article analyzes the differences, similarities, and scientific significance between these two main postgraduate doctoral types in order to give young dentists the ability to reflect on this distinction. 

(A) The regular doctoral degree, Dr. med. dent., involves an extensive literature review (narrative or systematic) and the completion of a dissertation, which can be submitted and does not have to be defended in front of a committee. This particular type of postgraduate doctoral degree does not require a complicated scientific research project or experimental laboratory or clinical work. Retrospective studies can be considered for this type of postgraduate doctoral degree. This type of postgraduate doctoral degree is the most common for students who want to focus mainly on a clinical career in private practice and want the postgraduate doctorate degree mainly for prestige purposes. The university degree “Zahnarzt” (in dentistry) or “Arzt” (in medicine) does not contain a “doctor” although it has the same validity as the DMD/DDS/MD degrees in English speaking countries. For this reason, many dental students choose a regular doctorate, as it is less complex and easier to obtain. An extensive independent research project and final defense examination are not required for this type of doctoral degree. The dissertation can be submitted once the project is completed, and after thorough review and acceptance by a committee, the candidate is granted the doctoral degree. The dissertation usually cannot exceed a final grade higher than “rite” or rarely a “cum laude”. The highest scores, such as “magna cum laude” (very good) and “summa cum laude” (outstanding), cannot be granted Table 2. The regular doctorate holders are encouraged but not required to publish the results of their project in a peer-reviewed journal.

(B) The research-intensive Dr. med. dent./Ph.D. equivalent is a traditional, individual doctorate, which includes additional extensive scientific research elements. Although there is still confusion if this postgraduate doctorate resembles the Ph.D.-equivalent degrees in English-speaking countries, it must be taken into consideration that this type of postgraduate doctorate contains all elements of a traditional, individual doctorate/Ph.D. as described by the German Federal Ministry of Education and Research on their official website and can be considered a Ph.D. equivalent internationally. The roots of confusion date back in history, especially in the field of dentistry. During the mid-19th century, dentistry was taught in the Faculty of Philology instead of the Faculty of Medicine and did not require a high school diploma as a prerequisite. Prior to 1900, a minority of dentists obtained their postgraduate doctorate Ph.D. degree in the USA, which was not offered in Germany at the time. On 8 June 1919, Baden was the first German Bundesland to introduce a doctorate in dentistry with great efforts and referred to it as Dr. chir. dent. The rest of the federal states (Bundesland) joined the new regulation, and the majority referred to the doctorate in dentistry as Dr. med. dent. During the past century, it became more popular among dentists to obtain a postgraduate doctoral degree, especially because it allows dental students to begin prior to graduation their dental studies and obtain the degree after graduation. The trend has been established in the past 50 years, and the prevalence of dentists obtaining a postgraduate doctorate has increased. Similar tracks exist today in the USA, such as combined master’s/Ph.D. programs, in which students start the programs before graduation. The main difference between the regular and the research-intensive (Ph.D. equivalent) doctoral degrees in Germany is the time-consuming, extensive scientific research, which involves long laboratory or clinical work hours (in the latter), efforts, and complexity of the projects, which can sometimes lead students to look for easier topics or even abandon their research projects completely [7]. If a doctorate candidate manages to handle the scientific load and master the complexity of the chosen project, they are encouraged to publish their work, as most of the independent scientific projects of doctorate holders result in publications in peer-reviewed journals. This is considered a requirement for a higher grade (“magna/summa cum laude”). Becoming a published author after graduating from medical/dental school allows postgraduate doctorate holders to gain a unique scientific experience as a baseline for a future academic and research career. 

However, there are some variations between the regular and research-intensive doctorates, in which elements of both blend, and distinguishing both types becomes challenging.

- When doctorate candidates start a research-intensive Dr. med. dent./Ph.D. equivalent and decide later to submit the dissertation without publishing their data and without a defense exam, due to lack of time, decrease in motivation, or other personal reasons, their doctorate will be considered a regular doctorate and will be granted a lower grade despite the scientific effort.
- When doctorate candidates start a research-intensive Dr. med. dent. and decide later to submit the dissertation after publishing their data without seeking a doctorate defense examination. Their doctorate then will be considered a regular doctorate and will be granted a lower grade despite the scientific effort. However, the published data may increase the final grade up to “cum laude” dependent on the candidate’s performance in the program.
- When a regular doctorate candidate decides to invest more time and effort, and their work ends in scientific publication in peer-reviewed journals, they can request a doctorate dissertation examination and can receive higher grades than “rite”, such as “cum laude”, and under further consideration and evaluation of the quality and scientific significance, it can be upgraded to literature research-intensive Dr. med. dent.; however, the lack of actual extensive experimental/clinical scientific research will not allow the highest notes and the consideration of a Ph.D. equivalent, as the highest notes, such as “magna-”or “summa cum laude”, and the scientific value of a Ph.D. equivalent are only considered for postgraduate doctorates that include extensive independent scientific research with significant contribution in sciences.

In general, the regular doctorate is considered to have a lower scientific value and oftentimes wrongfully considered a courtesy title. Candidates of this type of doctorate receive lower grades, as it requires less time and effort and does involves neither an independent, time-consuming scientific research project nor a rigorous doctorate defense examination in front of a committee.

## 6. Structured Doctorate/Ph.D. Programs 

This type of doctorate/Ph.D. program differs from traditional/individual doctoral research. These programs resemble the English-speaking countries’ academic model, where an academic team teaches and supervises a group of doctoral students. The program generally features a teaching curriculum that accompanies the Ph.D. interdisciplinary and promotes the acquisition of soft skills and additional qualifications. Unlike the individual doctorate model that can be freely structured to suit the individual research project, doctoral students enrolled in a structured postgraduate doctorate/(Ph.D.) program and their research proposals must fit in with an existing Ph.D. program. This frequently involves a didactic schedule and covers academic and scientific methods or soft skills, such as presentation techniques. 

## 7. Doctorate/Ph.D. Program in Cooperation with a Company 

These degrees are associated with research proposals, which can be performed in the research lab of companies and are granted by a university. The supervisor is always a faculty member, and the candidate performs the experimental part of research in the company facility and the scientific lab of a company. This type of postgraduate degree is very uncommon for dental and medical students.

## 8. German Dental Postgraduate Doctoral Degrees—The Good and the Bad

The variety of different avenues for obtaining a postgraduate doctoral education in Germany (or German-speaking countries, such as Switzerland) presents an obvious advantage compared to the academic model in English-speaking countries, as it offers flexibility and creativity within the decision making, Table 3. The traditional, individual doctorate, whether the regular type or the research-intensive Ph.D. equivalent, can give candidates the opportunity to pursue it at an institution, such as a university, while being employed in a company of private practice after graduation if they do not have a full-time job in the academic institution. This is a great option for new graduates who are financially challenged and have student loans, as it allows them to work in their field while working on their postgraduate program in their free time. Dental and medical students are also allowed to initiate the postgraduate research project part of the doctorate process during their undergraduate studies and work on these projects in their free time prior to graduation. It is no secret that medical and dental students spend most of their vacations and holidays/weekends working on their scientific projects [4,8].

Students who strive for clinical careers or private practice can choose between a regular or research-intensive doctorate. In contrast, students who want to pursue an academic career are encouraged to obtain the more scientifically significant Ph.D. equivalent doctorate. In both cases, students do not receive a salary for their research (doctoral) work. Additionally, there is no tuition for postgraduate doctoral degree programs at German public universities. Both facts are advantageous, as they exclude direct commitments regarding deadlines except for the 5-year completion from the start of the doctorate process, unless otherwise mentioned in the candidate doctoral contract. 

This freedom and flexibility, however, can sometimes be of disadvantage for young and inexperienced students, who can easily get overwhelmed and discouraged by the complexity of the scientific load, new information, and difficult, unfamiliar scientific methods, research, data collection, and analysis. This can sometimes lead to frustration and a lack of motivation, which is one of the reasons why not all doctorate candidates obtain a degree [7]. This flexibility encourages initiative, creativity, and strong organization skills. Personal drive, ambition, and commitment to sciences are the key elements for the candidates of these programs who want to succeed. Even though a research-intensive doctoral program/Ph.D. equivalent is very time-consuming and often complex, it remains the desired option for dental professionals interested in an academic career. This type of “natural selection” is a very common form of selection for graduates in German universities as education is free of tuition—and although accepted for study, graduation is not guaranteed for every student that matriculates. The same principle applies to postgraduate doctorates. Not every doctoral degree candidate obtains the degree in contrast to structured Ph.D. programs in English-speaking countries, where almost every registered Ph.D. student graduates due to the financial responsibilities they have (programs require tuition). Some German doctorate students abandon their projects and focus on their clinical careers due to the complexity of the independent scientific project, frustration, lack of time, and lack of motivation. The German university dental degrees obtained after graduation have been considered of equivalent value and significance as the DMD/DDS of the English-speaking countries, but German dental graduates are not called “doctor” without higher postgraduate education, and this distinction puts pressure on them to obtain higher doctorate degrees. They often decide to pursue the avenue of the regular doctorate, as it is not as time-consuming and complex as the research-intensive type.

## 9. The Postdoctoral Degree—The “Habilitationsschrift” (Dr. Habil.)

The main postdoctoral degree (after completion of the postgraduate Dr. med. dent. degree) in higher education presently in German-speaking countries is the “Habilitationsschrift” (second Ph.D.), which gives the distinction to physician-scientists to develop independent research projects and answer multiple scientific questions [9]. Additionally, there is verification for teaching qualifications and licensure (so-called academic freedom) in higher education with the Venia legendi. The candidates for this degree work in close contact with their mentees, who provide research data ready to publish in peer-reviewed, high-impact factor journals. In order to obtain this degree, there are specific criteria and a full-time commitment with teaching responsibility for a period of 4–6 years (after the specialty/residency training). There is no specific deadline for the completion of this program, and the decision is always made by the supervising professor, who evaluates the quality of the completed publications, teaching quality, and commitment to education. Additionally, there is a requirement of 12–16 qualified publications with the first or last authorship of the individual. The number of qualified publications varies among universities. This type of postdoctoral degree is called “cumulative” since the number of peer-reviewed publications is a key consideration for the degree [10]. There is a possibility, however, to complete a monography. For the specific existing dental specialties, such as oral/oral and maxillofacial surgery and orthodontics, it is required to complete the specialty before applying for the postdoctoral training. The evaluation occurs with relatively standardized criteria in order to bring some benchmarks to supervisors and candidates for these programs. The completion of this postdoctoral program, the Dr. habil. (Habilitationsschrift), takes place with a lecture (Colloquium) in front of a committee consisting of senior faculty members (Professors of Medicine and Dentistry), with an active discussion in the form of an evaluation followed by a lecture open to the community. Here, the candidate makes the commitment to continue (or not) his/her teaching activities and responsibilities to the institution [11]. If this is agreed, the individual acquires the title of “Associate Professor” in the specific institution and has the degree “Privat Dozent” (PD, or Priv. Doz.). The individual physician-scientist has the authority and license of institutional teaching and can continue independent research, including supervising Ph.D. students. This kind of postdoctoral degree exists in Switzerland and Austria but also in other European countries, such as Poland, Hungary, Finland, and Sweden, and gives the verification for independent, proficient level of teaching and clinical and research qualifications. The continued commitment to teaching, research, and clinical excellence gives the individual the chance to become an “APL” (“aussenplanmässig”) professor in the institution. There are requirements, such as the new publication list (after the Dr. habil. degree) of almost 12–16 peer-reviewed articles, the direct supervision of postgraduate students for the completion of a Dr. med. dent. degree (regardless of regular or research-intensive) and a period of a minimum of 2 years before submission of the application. The requirements are not the same in all universities because they are dependent on the educational regulations of the individual state. It is always advised to look at the websites of the universities for specific regulations. The requirements for assistant professors to become further associate professors at German medical faculties are very high. More than 88% of the associate professor regulations demand sufficient continuous performance in teaching and research with an adequate number of scientific publications [12], which makes a rigorous scientific preparation during the early stages of medical/dental studies imperative for deepening further scientific knowledge and proficiency, so these requirements can be met in the future [13].

## 10. Discussion

Both Germany and the United States of America have a long tradition of science and medical excellence. Although there are significant similarities in both systems, the approaches per se in undergraduate and postgraduate medical/dental education vary significantly. Even though the pathways for obtaining a medical/dental school title are different, the degrees in both countries are considered equivalent in terms of educational credit hours, level, and quality of education [14]. A similar trend is observed in postgraduate education, particularly in the postgraduate doctorate/Ph.D. degrees granted in both countries. The U.S. model offers strict structured doctorate/Ph.D. and integrated MD–Ph.D./DDS–Ph.D. programs, respectively, whereas Germany offers more flexibility and freedom of choice, offering a few different pathways for postgraduate doctorates, most of which include the most significant elements of a Ph.D. degree, as it is known worldwide. The lack of understanding of the different postgraduate doctoral and postdoctoral qualifications is creating a disbalance and inequity among German academic degree holders and their academic environment, especially when they want to pursue an academic career in English-speaking countries. There is a discrepancy in the perception among German and international academics regarding the dental postgraduate levels of education. The lack of a thorough review and analysis of the differences and similarities as well as the scientific value among the different doctorates creates confusion and often puts doctoral and postdoctoral degree holders in an unfavorable position where they do not receive the credentials they deserve. It is essential for international academic and education evaluation institutions, such as ECE—Educational Credential Evaluators—and WES—Educational Credential Evaluators—to have a clear conception of the variety of options and the characteristics of each postgraduate level of education. These evaluation services have the power to categorize education and academic degrees, and their assessments need to reflect the exact scientific value. Educational evaluation services are not always familiar with the different options for postgraduate doctorates in Germany and the even higher academic level of the postdoctoral doctorate (“Habilitation”), their characteristics, and scientific values. Oftentimes, the educational evaluation provided by international evaluation services is not accurate, as they consider the German dental/medical postgraduate doctoral degrees of a value of a master’s degree, which is academically not correct. There are numerous differences between a postgraduate master’s degree and a doctoral degree and the appeal for more transparency in the educational evaluation process. According to the Federal state report about Young Scientists 2017 (Bundesbericht Wissenschaftlicher Nachwuchs, 2017), there has been confusion about the relevance, significance, and implementation of the different postgraduate doctorate types in all scientific areas (https://portal.unikoeln.de/fileadmin/home/amgc/PDF/buwin_2017.pdf (accessed on 25 September 2021)). After a thorough review of existent data from the main sources of information such as the German Federal Ministry of Education and Research and the DAAD, the Deutscher Akademischer Austauschdienst (German Academic Exchange Service), the authors suggest four types of postgraduate doctorates available in Germany, the first two of which are mainly chosen by dental and medical students. Furthermore, the authors emphasize the reasons for choosing one or the other, as each postgraduate doctorate has a different scientific value—the regular doctoral degree being preferred by students who are mainly interested in private practice and a clinical career, whereas the research-intensive doctorate (Ph.D. equivalent) is mostly obtained by professionals interested in growing academically in Germany as well as internationally.

Research-intensive doctorates in medicine and dentistry should be considered equivalent to the Ph.D. degrees in English-speaking countries, as they contain most of the elements required, representing the same process and approach as explained on the official internet website of the German Federal Ministry of Education and Research. The research-intensive doctorate requires self-discipline, motivation, commitment, and passion for science and is often perceived as very difficult and overwhelming. In some cases, doctorate candidates do not complete their project because of this fact. Some reasons why students give up are lack of supervision (mainly for scientific projects at the Departments of Medicine), difficulty in recognizing any progress (lack of successful experiments or significant data), inappropriate timing, and unacceptable burden of work, according to Diez et al. [7]. Further factors contributing to the lack of interest in research among undergraduate and postgraduate medical students are uncooperative faculty members, funding issues, and family constraints (married vs. unmarried students) [15,16]. Postgraduate student well-being and well-established rapport and communication are other important aspects for the successful completion of scientific research projects [17,18]. Having one supervisor has the advantage of individual supervision and direct guidance, which is mostly observed in the research-intensive type of doctorate. Overall, despite some negative aspects, German postgraduate doctoral degree programs are still very attractive for young and older graduates. The official website of the German Federal Ministry of Education and Research is one of the greatest and most trusted online sources of information. It provides links and further details and explains why German postgraduate doctorates are so desired among German and international graduates. However, there are some pros and cons between the different German doctorates; the degree Dr. med. dent. in dentistry (Dr. med. in medicine, respectively) is equal independent of whether the doctorate is regular or research-intensive. The only distinction is the scientific value and the final grade of the doctorate: “rite” or “cum laude” for the regular vs. the possibility for magna and summa cum laude for the research-intensive doctorate type. The only official way to distinguish both is to analyze the diploma, which contains the final grade and gives information regarding whether the doctorate degree was defended in front of a committee. The latter requires consistent motivation, persistence, exceptional organizational skills, and independence, as the complexity of the scientific approaches is included, and unexpected outcomes in experimental/clinical approaches might discourage the candidates [19]. This type of degree, however, is still very desired by young graduates who are interested in academic and teaching careers, as it offers extensive scientific research experience and gives the candidates great opportunities for academic growth.

We noticed the lack of transparency in regards to the scientific value of the different doctorate types not only on an international level. Numerous German Universities are not completely aware of the similarities between the individual research-intensive doctorate in dental fields and a conventional Ph.D. in other countries. New Ph.D. programs are emerging and have the purpose of accommodating diverse interests and avoid differences in the education of German doctoral degree holders internationally. Re-consideration of the old academic system is needed to re-adjust and follow the academic trends internationally and to include possibilities for young dentists who decide later in life to pursue a career in academia. These young dentists are oftentimes overwhelmed and lack a clear vision and plan. Further detailed information can also be found on the official website of the DAAD, the Deutscher Akademischer Austauschdienst (German Academic Exchange Service), about the different Ph.D. options (https://www.daad.de/en/study-and-research-in-germany/phd-studies-and-research/ (accessed on 25 September 2021)). Together with the official website of the German Federal Ministry of Education and Research, the DAAD (Deutscher Akademischer Austauschdienst, German Academic Exchange Service) is one of the main online sources for information about higher education in Germany, academic scientific research for postgraduate doctoral degrees, as well as postdoctoral academic degrees and opportunities provided up to date. The DAAD is also the main source of academic guidance for graduates interested in academic scientific research in Germany and abroad (https://www.daad.de/de/studieren-und-forschen-in-deutschland/promovieren-und-forschen/promovieren/phdgermany-info/ (accessed on 25 September 2021)). In the past, only the first two types of doctorates were common in the field of medicine and dentistry. The trend toward the academic model of English-speaking countries began to emerge in recent years, and some German medical and dental universities are beginning to consider offering structured Ph.D. after graduation or integrated DDS/Ph.D. or MD–Ph.D. programs within the study of dentistry (or medicine). The “gold standard” remains the traditional, individual doctorate, either the regular or research-intense/Ph.D. doctorate degree. A lack of familiarity with the different types of postgraduate doctorates of German doctorate holders creates a great deal of confusion and oftentimes underestimates an individual’s academic achievements of doctorate holders. (Figure 2).

Another important aspect of some of the ongoing academic conflicts is the dynamic modern life, which requires flexibility and efficiency in postgraduate education. The strict, structured Ph.D. programs might eventually become obsolete and fully replaced by integrated MD–Ph.D./DDS–Ph.D. programs in the USA, following the example of flexibility and more freedom in scientific research presented by the German academic model.

## 11. Conclusions

The different avenues for obtaining postgraduate dental doctorate/Ph.D. degrees in Germany make the process of evaluation of doctorate degrees (Dr. med. dent.) a very challenging topic, especially for international employers, academics, and educational evaluation services. Understanding the scientific value and overall significance of each postgraduate doctorate type is crucial for academic recognition. German doctorate holders demonstrate competency in performing a research proposal and answer a scientific question under supervision. In contrast, the next level in the academic hierarchy—the postdoctoral degree (Dr. habil.)—is proof of teaching qualifications, including supervision of doctorate/Ph.D. students, academic freedom, and proficiency in independent research. The German academic training and, in general, the postgraduate education today is very comprehensive and well-organized and provides opportunities for academic growth as well as a solid foundation of knowledge for scientists. Young graduates should consider the various options of education across the globe to make their decision based on their goals to become leaders in academic dental education. Without a doubt, both Germany and the USA offer a high quality of education, and the requirements for promotion in the academic hierarchy in both countries are rigorous. Therefore, it is important for young clinicians who are interested in a career in academia to begin with their first research projects early in their study [20]; this is the mutual point where the flexibility of the German academic model meets the efficiency of the integrated MD/Ph.D. and DDS/Ph.D. programs in the USA, respectively.

## Figures and Tables

**Table 1:** Countries with the most postgraduate doctoral holders (OECD 2016).

Country	Postgraduate Doctoral Holders
United States	69,525
China	55,151
Germany	29,303
Russia	27,212
United Kingdom	27,009
India	25,095
Japan	15,804

**Table 2:** Possible overall grades for German postgraduate doctoral degrees.

Latin	English
“Summa cum laude”	Outstanding performance
“Magna cum laude”	Very good performance
“Cum laude”	Good performance
“Rite”	Satisfactory performance
“Non-sufficit”	Unsatisfactory performance

**Table 3:** Differences and similarities between all four different doctorate types in Germany related to postgraduate medical and dental doctorates. * This type is rarely seen in medicine/dentistry.

	Traditional, Individual Postgraduate Doctorates in Dentistry		Structured Ph.D.	Ph.D. in Cooperation with a Company *
	Regular Doctorate— Dr. med. dent.	Research-Intense (Ph.D. Equivalent) Dr. med. dent.	Dr. med. dent.	Dr. med. dent.
**Supervisors**	1	1	multiple	multiple
**Choose topic alone**	YES	YES	given	given
**Independent Experimental/** **Clinical Scientific Research**	NO	YES	YES	YES
**Didactic element**	NO	NO	POSSIBLE	Company position
**Autonomy**	YES	YES	LESS	LESS
**Flexibility**	YES	YES	LESS	LESS
**Oral doctoral defense examination**	NO	YES	YES	YES
**Potential for Publication**	Not necessary	Encouraged/Required	YES	NO

## Data Availability

Not applicable.

## References

[1] Dewey M. (2003). Students’ evaluation of research during medical studies: Medical dissertation in Germany. Med. Educ..

[2] Sorg H., Krämer R., Grieswald C., Schwab C.G.G., Paprottka F.J., Steiert A.E., Tilkorn D.J., Hauser J. (2016). The medical dissertation in Germany: A quantitative analysis of promotion regulations in medical faculties. Chirurg.

[3] Kuhnigk O., Reissner V., Böthern A.M., Biegler A., Jüptner M., Schäfer I., Harendza S. (2010). Criteria for the successful completion of medical dissertations—A multicenter study. GMS Z. Med. Ausbild..

[4] Altunbas A., Cursiefen C. (1998). Research activities of medical students in Germany using as an example the Würzburg University Clinic. Dtsch. Med. Wochenschr..

[5] Diez C., Arkenau C., Meyer-Wentrup F. (2000). The German medical dissertation—Time to change?. Acad Med..

[6] Brass L.F., Akabas M.H. (2019). The national MD-PhD program outcomes study: Relationships between medical specialty, training duration, research effort, and career paths. JCI Insight.

[7] Diez C., Arkenau C., Meyer-Wentrup F. (2000). Why German medical students abandon dissertations. Educ. Health.

[8] Cursiefen C., Altunbas A. (1998). Contribution of medical student research to the Medline-indexed publications of a German medical faculty. Med. Educ..

[9] Knobloch K., Sorg H., Vogt P.M. (2012). Postdoctoral qualification regulations of medical faculties in German universities. A comparison of 1998 and 2010. Chirurg.

[10] Weineck S.B., Koelblinger D., Kiesslich T. (2015). Medical habilitation in German-speaking countries: Quantitative assessment of content and elaboration of habilitation guidelines. Chirurg.

[11] Sorg H., Krämer R., Grieswald C., Schwab C.G., Thönnes S., Reinke J.M., Hauser J., Tilkorn D.J. (2016). Assessment of the significance and the requirements of medical postdoctoral lecture qualifications in Germany by the assessment committees. Z. Evid. Fortbild. Qual. Gesundhwes..

[12] Sorg H., Knobloch K. (2012). Quantitative evaluation of the requirements for the promotion as associate professor at German medical faculties. GMS Z. Med. Ausbild..

[13] Nagelschmidt M., Bergdolt K., Troidl H. (1998). Evaluation of qualification regulations for medical faculties of German universities and recommendations for standardization. Chirurg.

[14] Zavlin D., Jubbal K.T., Noé J.G., Gansbacher B. (2017). A comparison of medical education in Germany and the United States: From applying to medical school to the beginnings of residency. Ger. Med. Sci..

[15] Saeed I., Khan N.F., Bari A., Khan R.A. (2018). Factors contributing to the lack of interest in research activities among postgraduate medical students. Pak. J. Med. Sci..

[16] Sheikh A.S., Sheikh S.A., Kaleem A., Waqas A. (2013). Factors contributing to lack of interest in research among medical students. Adv. Med. Educ. Pract..

[17] Schmidt M., Hansson E. (2018). Doctoral students’ well-being: A literature review. Int. J. Qual. Stud. Health Well-Being.

[18] Conn V.S., Zerwic J., Rawl S., Wyman J.F., Larson J.L., Anderson C.M., Fahrenwald N.L., Benefield L.E., Cohen M.Z., Smith C.E. (2014). Strategies for a successful PhD program: Words of wisdom from the WJNR Editorial Board. West. J. Nurs. Res..

[19] Young S.N., Vanwye W.R., Schafer M.A., Robertson T.A., Poore A.V. (2019). Factors Affecting PhD Student Success. Int. J. Exerc. Sci..

[20] Kanwar R.S. (2010). Skill set development of doctoral and post-doctoral graduates in life sciences. Commun. Agric. Appl. Biol. Sci..

